# Fish taxonomic, functional, and phylogenetic diversity and their vulnerabilities in the largest river in southeastern China

**DOI:** 10.1002/ece3.7945

**Published:** 2021-07-24

**Authors:** Li Lin, Weide Deng, Xiaoxia Huang, Bin Kang

**Affiliations:** ^1^ College of Fisheries Ocean University of China Qingdao China; ^2^ Department of Oceanography National Sun Yat‐Sen University Kaohsiung Taiwan; ^3^ Henry Fok College of Biology and Agriculture Shaoguan University Shaoguan China; ^4^ Key Laboratory of Atmospheric Environment and Processes in the Boundary Layer over the Low‐Latitude Plateau Region School of Earth Science Yunnan University Kunming China; ^5^ Key Laboratory of Mariculture (Ocean University of China) Ministry of Education Qingdao China

**Keywords:** alpha diversity, beta diversity, congruence, freshwater, systematic conservation planning

## Abstract

1. Freshwater biodiversity is currently under multiple threats. Conservation of freshwater fish biodiversity needs to be prioritized because natural conservation resources are always limited.
2. Samples were collected at 24 sites in the Min River, the largest basin in southeastern China. Taxonomic, functional, and phylogenetic diversity were analyzed. Biodiversity vulnerability was measured by removing one species each time out of the community with replacement.
3. Results suggested that hotspots for taxonomic and phylogenetic diversity were located at two impounded sites, while for functional diversity were those sites with no upstream dams. Little congruence was observed between taxonomic, functional, and phylogenetic diversity. Fragmentation of river network connectivity caused by dams was a significant factor affecting the biodiversity patterns. Beta turnover was the driving component for beta diversity, indicating that biodiversity dissimilarity along the river was mostly explained by environmental sorting. Fifteen out of 16 species that contributed the most to different facets of biodiversity were mostly endemic, either they had distinctive functional traits or they were the most prevalent species. Sites with the highest diversity vulnerability were characterized by these distinctive species. Functional diversity was more vulnerable to species loss comparing with the other two biodiversity facets.
4. Prioritizing those biodiversity hotspots, sites with extreme functional vulnerability, and those distinctive endemic species which contributed the most to biodiversity vulnerability is suggested in the Min River. The study found evidence that congruence among different facets of biodiversity is hard to achieve, and functional diversity is the most vulnerable in a freshwater system fragmented by intensive dam constructions. This work will help to develop systematic conservation planning from the perspective of different biodiversity facets.

## Graphical abstract

Fish functional diversity is more vulnerable than taxonomic and phylogenetic diversity in a freshwater riverine system segmented by an intensive distribution of dams.

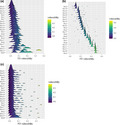

## 1 INTRODUCTION

Global biodiversity is being threatened by multiple factors such as anthropogenic interference, climate change, and invasive alien species (Brook et al., 2008). Species in freshwater ecosystems are more seriously threatened than in terrestrial and marine ecosystems. According to recent estimates, the global freshwater vertebrate population has declined by 84% since 1970, while the terrestrial vertebrate population has declined by 40% and the Marine vertebrate population has declined by 35% (Bongaarts, 2019). Although freshwater fish alone account for a quarter of all living vertebrate species, over 30% of them are experiencing small population size, habitat loss, or habitat fragmentation (Carrizo et al., 2013). Thus, freshwater biodiversity conservation becomes more urgent and imminent than ever before.

Biodiversity is unevenly distributed and is threatened by multiple threats throughout the world. Meanwhile, conservation budgets are always limited, so spatial prioritization is essential to achieve conservation objects and ensure every penny spent is worthy (Brooks et al., 2006). Spatial prioritization refers to quantitative techniques that provide policy‐makers and managers with spatial information to determine what strategies should be adopted to address environmental problems, identify the most valuable regions, ecosystems, and species, and maximize the effectiveness of financial resources (Mendoza‐Ponce et al., 2020).

It is increasingly recognized that biodiversity is constituted by multifaceted components, including taxonomic diversity (TD), functional diversity (FD), and phylogenetic diversity (PD), each representing different information and ecological value. TD refers to the number and relative abundance of species in a community (Magurran, 2004). Classically, conservation actions from the perspective of TD focused on rare species with high conservation value (Zhang et al., 2021), or regions with large species richness (Vilar et al., 2017). However, TD alone conveys little information regarding the function or evolutionary history of species. Functional diversity (FD) refers to those components of biodiversity that influence how an ecosystem operates or functions (Mouchet et al., 2010). Conservation of FD often concentrated on species with functional rarity and distinctiveness (Grenie et al., 2018), or places bearing more functional groups (Lamothe et al., 2019). Phylogenetic diversity (PD) is typically defined as the sum of the branch lengths of the minimum spanning path joining a set of taxa on a phylogenetic tree (Faith, 1992). Conservation biologists are interested in PD because maximizing PD is assumed to conserve the most genotypes or phenotypes of different phylogenetic taxa in a community (Veron et al., 2019). Many conservation practices try to seek congruence of TD, FD, and PD, aiming to balance the protection of different biodiversity facets at the same time (Doxa et al., 2020; Strecker et al., 2011; Wong et al., 2018).

Over the past decades, different efforts have been dedicated to prioritizing diversity conservation, and most of them fall into two frameworks: irreplaceability and vulnerability (Brooks et al., 2006). The irreplaceability of a site has been defined in two ways: (a) the likelihood that a site will be required to realize a given set of conservation targets; and (b) the extent to which these targets can be achieved even if the area is lost. Prioritizing biodiversity “hotspots” intrinsically belongs to this category. The logic is that the more biodiversity one site processes, the more irreplaceable this site is, because if it is not protected, more biodiversity will be lost (Ferrier et al., 2000; Mendoza‐Ponce et al., 2020; Pressey et al., 1994). Species do not contribute equally to ecosystem functioning (Mouillot et al., 2013), so another popular alternative measure of irreplaceability is species endemism, such as birds or plants endemism (Alvarez‐Alvarez et al., 2020; Prieto‐Torres et al., 2018).

The vulnerability of biodiversity is defined in several terms (Mendoza‐Ponce et al., 2020): (a) Exposure is defined as the degree, duration, and extent to which a system or a part of it is in contact with harm; (b) sensitivity is recognized as the susceptibility of an element to be harmed; (c) adaptive capacity refers to the ability to adjust to current or future conditions (IPCC, 2014; Mendoza‐Ponce et al., 2020). Mostly, the vulnerability is measured by habitat loss or fragmentation under climate change, land‐cover/use‐change, species invasion, and other anthropogenic interference (LeMoine et al., 2020; Maerz et al., 2019; Penaluna et al., 2015). Another direct metrics is to measure the impact of species loss (Chua et al., 2019; Pool et al., 2014).

Conservation efforts in freshwater ecosystems should also consider the effects of dendritic river networks because river network connectivity affects fish species sorting, dispersal dynamics, habitat availability, nutrition, and trophic dynamics (Shao et al., 2019). External factors associated with human activities, such as hydropower utilization and dam construction, create frequent and severe disruptions for natural river flows and significantly alter hydrological connectivity in river networks (Arantes et al., 2019; Herrera‐Perez et al., 2019). Impacts of these factors on freshwater fish biodiversity therefore need to be considered during the development of systematic conservation.

The Min River, ranging from 116.38°E to 119.72°E and 25.38°N to 28.32°N, is the largest basin in southeastern China and plays an important role in social, environmental, and economic development (Editorial Committee of Fujian Province Annals, 1999; Zhu, 2007). Over 150 fish species were inhabiting this basin in the 1970s, covering 99 genera, 31 families, and 14 orders (Lian, 1988; Zhu, 1984). This basin is also inevitably subjected to biodiversity crisis as a result of climate changes, species invasion, and anthropogenic activities like pollution, dam construction, and land‐cover/use changes in the past decades (Liu et al., 2009; Tang et al., 2013; Zhang et al., 2003). Biodiversity conservation in this basin becomes urgent and imperative. Although studies on fish communities in the Min River began in the early 1940s (Nichols, 1944), there have been scarce researches reporting fish diversity conservation in the basin to date. Therefore, based on a field investigation, this study evaluated the biodiversity of the Min River from multiple biodiversity facets and focused on two primary issues: (a) spatial patterns of biodiversity in the Min River, and (b) biodiversity hotspots, congruence of different biodiversity facets, and biodiversity vulnerability in terms of species loss. To our best knowledge, this is one of the first times that concerns riverine fish diversity conservation in subtropical areas in China and should provide important implications for systematic conservation in the Min River.

## 2 METHODS

### 2.1 Study region and sampling

**FIGURE 1:**
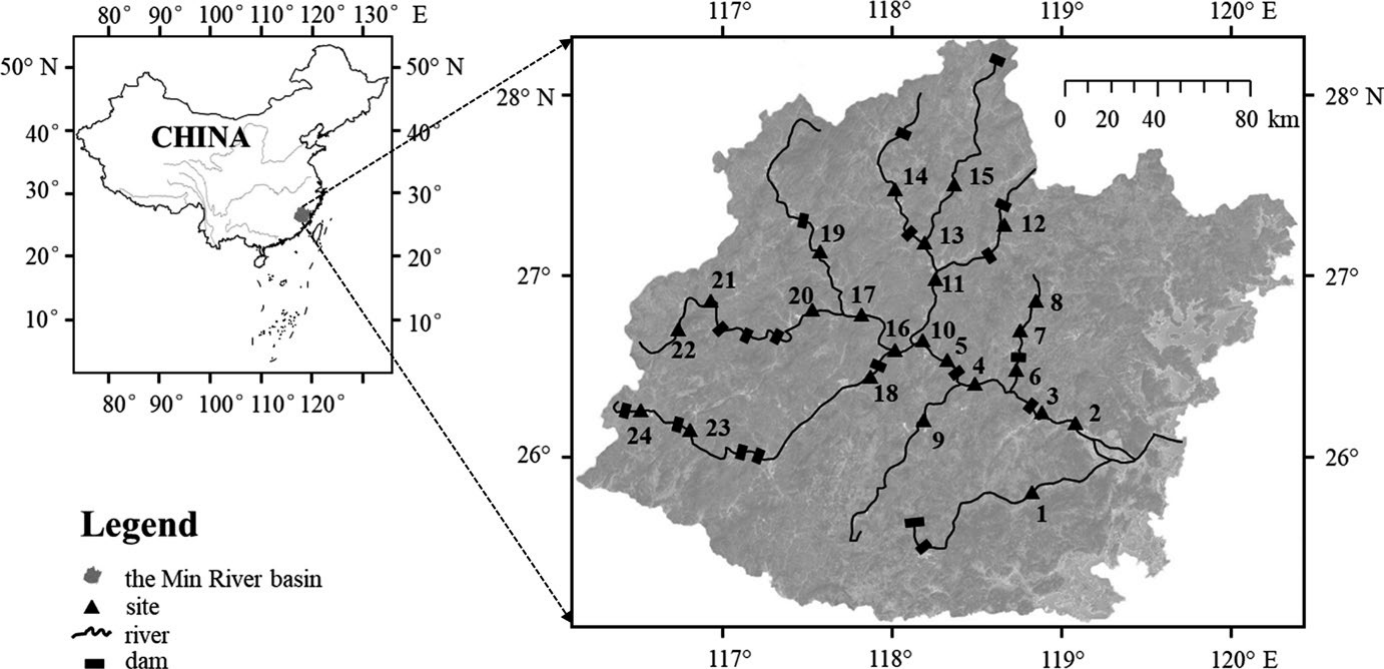
Sampling sites and distribution of dams from the upstream to the lower reach of the Min River, the largest river in the southeast of China. The river runs through Fujian Province, China

Sampling sites were distributed across the Min River (Figure 1). Information about dams in the basin was recorded in the field and partly extracted from Fujian Province Annals: Water Conservancy (Editorial Committee of Fujian Province Annals, 1999). Fishes were collected at 24 sampling sites in May 2015 from the upstream to the lower reach of the river according to its dendritic structure by the same means of electricity stunning under the permission and supervision of the local governance (backpack electrofishing unit, Model: CWB‐2000 P, Yufengda, China; 12‐V import, 250‐V export). To guarantee the representativeness of the samples and while protecting individuals, these electrofishing passes were conducted using a uniform sampling effort by the same four persons, with approximately 30 min of sampling time for each 50 m segment according to local fishers’ knowledge. Fishes were identified to species, measured, and returned to the sampling sites if alive.

Both abundance and presence/absence data were compiled. Abundance data were used for alpha TD and FD calculations. Presence/absence data were applied to PD and beta diversity calculations. Original taxonomic data were revised according to the records in FishBase to avoid invalid species, synonyms, and homonyms (Froese & Pauly, 2019). Overall, 82 species and 1762 individuals were collected and subjected to basic morphological traits measurement in situ (Table S1).

### 2.2 Dimensions of biodiversity

The interpretation of biodiversity has long been controversial. An individual diversity index is not “true” biodiversity at all (Jost, 2006), but each index can reflect certain information of the communities of interest (Bandeira et al., 2013; Chao et al., 2014; Faiths et al., 2008; Purvis & Hector, 2000). For alpha diversity, Shannon index (Shannon) was applied as a surrogate for TD because it is most commonly used, and it accounts for the relative importance of species abundance in a given environment (Magurran, 2004). Similarly, Rao's Quadratic Entropy index (RaoQ) was applied for FD because it represents a mix between functional richness and functional divergence (Mouchet et al., 2010). Finally, Faith's index (PDfaith) was applied for PD because it directly links to species evolution history (Faith, 1992). Since evenness and richness are complementary, indices reflecting evenness of biodiversity were also used. These indices included Shannon Evenness (SEve) (Magurran, 2004), Functional evenness (FEve) (Villeger et al., 2008), and Phylogenetic Species Evenness (PSEve) (Helmus et al., 2007).

For beta diversity, Sorenson dissimilarity family indices were applied because algorithms of these indices for TD, FD, and PD are analogous. Beta Sorensen (*β*_sor_) measures the total taxonomic, functional, or phylogenetic dissimilarity between two communities. Taken *β*_sor_ for TD, for example, it is formulated as:

(1) $$\beta_{\text{sor}}=\frac{b+c}{2a+b+c}$$

where a is the number of species common to both communities, b is the number of species that occur in the first community but not in the second, and c is the number of species that occur in the second community but not in the first (Baselga, 2010). Analogously, *a*, *b*, and *c* in this formula should be considered as functional traits for FD or phylogenic branches for PD (Leprieur et al., 2012; Villeger et al., 2013).

Turnover (*β*_sim_) and nestedness (*β*_nes_) are basically two constitutional components of the total beta diversity, that is, *β*_sor_. Turnover designates the replacement of some species by others as a consequence of environmental sorting or spatial and historical constraints, whereas nestedness implies a nonrandom process of species loss as a consequence of any factor that promotes the disaggregation of assemblages (Baselga, 2010).

Ten functional traits were compiled to quantify the functional space (Comte et al., 2014; Gatz, 1979; Villeger et al., 2013). Biological interpretations for these traits are shown in Table 1. These traits were selected based on three reasons: (a) Available information was retained as much as possible to depict functional space as intact as possible, (b) these derived traits reflect food acquisition, mobility, habitat preference, and life cycle of the fish species, and (c) all these traits are relevant to their living conditions in a freshwater system. These functional traits belong to the following functional groups: feeding habit, trophic, swimming capability, habitat preference, and life cycle.

**TABLE 1:** Ten functional traits applied for functional diversity analysis

Functional trait	Functional group	Type	Values	Biological interpretation
Relative head length	Feeding habit	Continuous	Hl/Sl	High values may indicate fish able to feed on relatively larger prey (Watson & Balon, 1984).
Relative eye size	Feeding habit	Continuous	Ed/Hd	Visual acuity, relating to prey detection (Gatz, 1979).
Relative snout length	Trophic	Continuous	Snl/Hl	The length of the snout affects a variety of trophic and sensory capabilities, influencing the abilities of fishes to detect and acquire prey (Toussaint et al., 2016).
Relative head depth	Trophic	Continuous	Hd/Bd	High values indicate deeper heads. Head depth plays a variety of roles in the sensory and trophic capabilities of a fish. Deep heads may also affect the hydrodynamics of a fish, increasing maneuverability (Toussaint et al., 2016).
Relative pectoral fin length	Swimming	Continuous	PecFl/Sl	Pectoral fin length is assumed to increase as a function of amount of low‐speed maneuvering in the behavior of fish (Watson & Balon, 1984).
Swimming factor	Swimming	Continuous	CPd/CFd	Hydrodynamics. Caudal propulsion efficiency through reduction of drag (Villeger et al., 2013).
Relative body depth	Habitat preference	Continuous	Bd/Sl	Relative body depth is assumed to be inversely related to habitat water velocity and directly related to capacity of making vertical turns (Gatz, 1979).
Rheophily	Habitat preference	Categorical	rheophilic, limnophilic, eurytopic	Prefers fast flows (rheophilic), slow flows (limnophilic), or adapted for a wide range of flow types (eurytopic) (Pool et al., 2014).
Position of the water column	Habitat preference	Categorical	benthic, benthopelagic, pelagic	Benthic; benthopelagic; pelagic (Pool et al., 2014).
Life span	life cycle	Categorical	<=10; 10–20; >20	Maximum life span in years (Pool et al., 2014).

Note

The continuous functional traits were calculated by the basic morphological traits. The categorical traits were extracted from “Fishes in Fujian Province” and FishBase.

Abbreviations: Bd, Body depth; CFd, Caudal fin depth; CPd, Caudal peduncle depth; Ed, Eye diameter; Hd, Head depth; Hl, Head length; PecFl, Pectoral fin length; Sl, Standard length; Snl, Snout length.

Continuous functional traits were calculated in situ in compliance with the FishBase manual (Froese & Pauly, 2019). Quantification of these traits is shown in Figure S1. Categorical traits including rheophily, position of the water column, and maximum life span were extracted from “The Fish of Fujian Province” (Zhu, 1984), “Chinese Fauna: Osteichthyes” (Chen, 2002), and FishBase (Froese & Pauly, 2019). Principle coordinate analysis (PCoA) extracted the first five dimensions which explained a 72% cumulative variance of these ten traits for further FD analysis.

Cytochrome B (CYTB) and cytochrome oxidase I (COI) gene sequences were obtained from the GenBank database on NCBI website (Gene, 2019) and concatenated. Firstly, the executable programs “Clustal W 2.0” and “MUSCLE 3.8” were comparatively applied for alignment to make sure the sequences were properly aligned (Edgar, 2004; Larkin et al., 2007). Then, the Akaike information criterion (AIC) was used to select the optimal parameters for the Most Likelihood (ML) method (Posada & Crandall, 2001). The substitution model “GTR + I + Γ” was the one that best explains the empirical data in terms of AIC (Figure S2), where “GTR” designates the general time‐reversible model; meanwhile, "+I" and "+Γ " indicate that proportion of variable size and that gamma rate parameter get optimized in the GTR model, respectively. Finally, the executable program “PhyML 3.1” was invoked for ML phylogenetic tree construction with R (Guindon et al., 2010).

Moran's I coefficient was used to detect spatial autocorrelation of TD, FD, and PD at the confidence level of 0.99 (Gittleman & Kot, 1990), while Lee's *L* test was used to detect bivariate spatial autocorrelation between them (Lee, 2004).

### 2.3 Spatial pattern of river system

Two classic indices are often applied to measure river network connectivity, including Strahler order (Order) (Arthur, 1957) and downstream link (Dlink) (Osborne & Wiley, 1992). Three categorical surrogates for river network connectivity were applied in this work, including Order, 2‐based logarithm of Dlink (log_2_Dlink), and the existence of dams (Dam). (a) For Order, the smallest finger‐tip tributaries originating from the water source are designated as Order 1; then, the confluence of two Order 1 streams formed an Order 2, furtherly two Order 2 streams joined together to form an Order 3 and so forth. (b) For log_2_Dlink, the magnitude of a link is firstly defined, which is the number of first‐order segments upstream of a given point on a channel; then, the Dlink at any point is the magnitude of the link below the next downstream confluence, finally a 2‐based logit‐transformation was performed for Dlink to divide different sampling sites into comparative categories. (c) For Dam, three categories were classified, including the existence of an upstream but no downstream dam, upstream and downstream dams, and a downstream but no upstream dam. Multivariate analysis of variance (MANOVA) was performed to test the impact of these three variables on TD, FD, and PD.

### 2.4 Calculation of biodiversity vulnerability

One individual species was removed out of the community each time, and the differences in the biodiversity were calculated before and after. This process was iterated with replacement until all the species had been removed once. The difference equaled the deduction of the biodiversity before and after the species had been removed from the system. The deduction of biodiversity was used as a measurement of biodiversity vulnerability (Heilpern et al., 2018; Midgley et al., 2002; Pool et al., 2014).

All geographic maps were drawn in ArcGis 10.0. All calculations and analysis were performed in R 3.6.1 (R Core Team, 2019) and executed under Windows 10 platform. Main packages and related functions are listed in Table S2.

## 3 RESULTS

### 3.1 Patterns of alpha biodiversity

**FIGURE 2:**
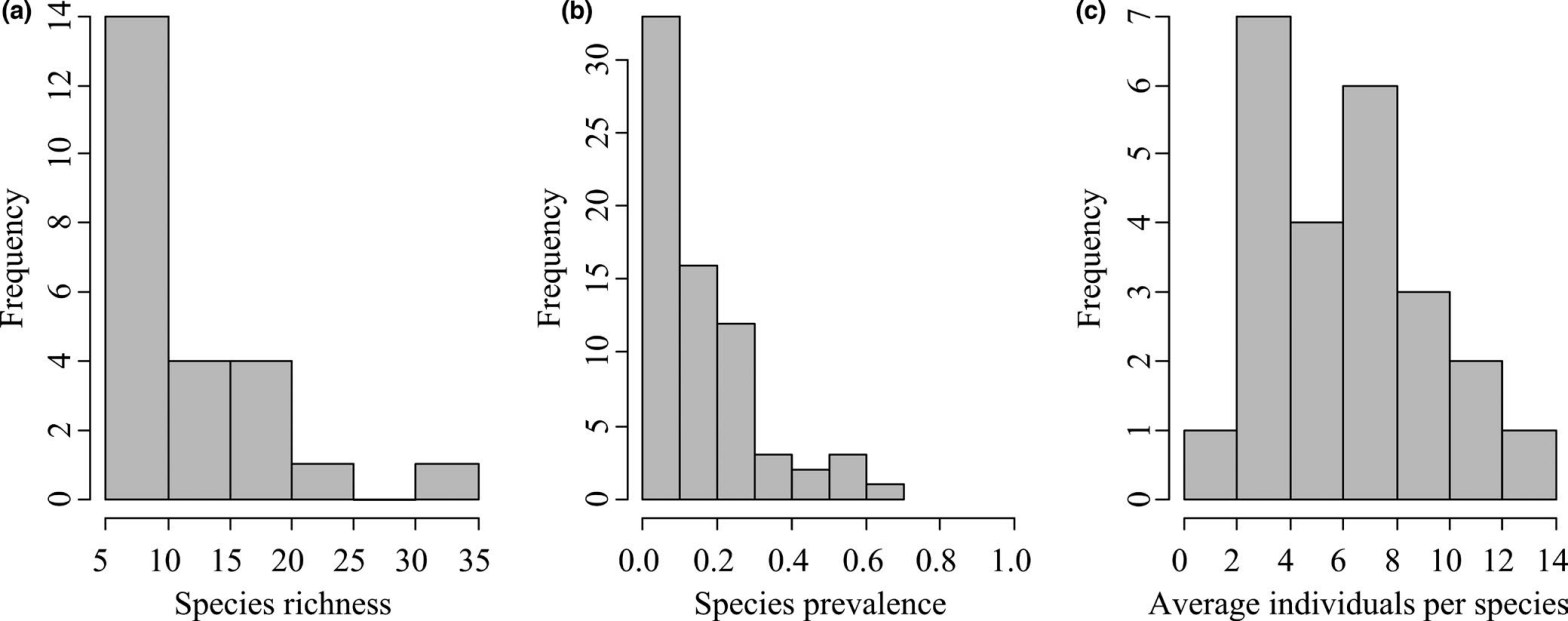
Histograms of (a) species richness, (b) species prevalence, and (c) average individuals per species in the Min River based on field investigation. Species prevalence is calculated as the total occurrence of each species divided by the total number of sampling sites. Average individuals per species are calculated as the total number of collected individuals divided by species richness

Species richness at most sampling sites (14 sites out of 24) ranged from 5 to 10, and most species had a low prevalence (occurrence divided by number of sampling sites) (Figure 2). The highest species richness was 35 at Site 21, while the lowest was 6 at Site 9. The highest average individual per species was 12.67 at Site 14, while the lowest was 1.14 at Site 17.

Moran's I coefficient confirmed that no significant spatial autocorrelation was detected for TD, FD, or PD (Table 2, *p* > .01) and Lee's *L* test detected no bivariate spatial autocorrelation (Table 3, *p* > .01), indicating that observations at these 24 sites were independent.

**TABLE 2:** Moran's I autocorrelation coefficient for different facets of biodiversity

Moran's I	Shannon	RaoQ	PDfaith
Observed	0.06	0.10	0.08
Expected	−0.05	−0.05	−0.05
*SD*	0.07	0.07	0.06
*p* value	.12	.03	.04

Note

Indices used are Shannon index (Shannon), Rao's Quadratic Entropy index (RaoQ), and Faith's PD index (PDfaith).

**TABLE 3:** Lee's *L* test for bivariate spatial correlation between different biodiversity facets

Variable 1	Variable 2	Lee's *L* statistics	Expected	*p* value
Shannon	RaoQ	0.38	0.23	.36
Shannon	PDfaith	0.98	0.86	.69
RaoQ	PDfaith	0.12	0.01	.33

Note

Indices used are Shannon index (Shannon), Rao's Quadratic Entropy index (RaoQ), and Faith's PD index (PDfaith).

**FIGURE 3:**
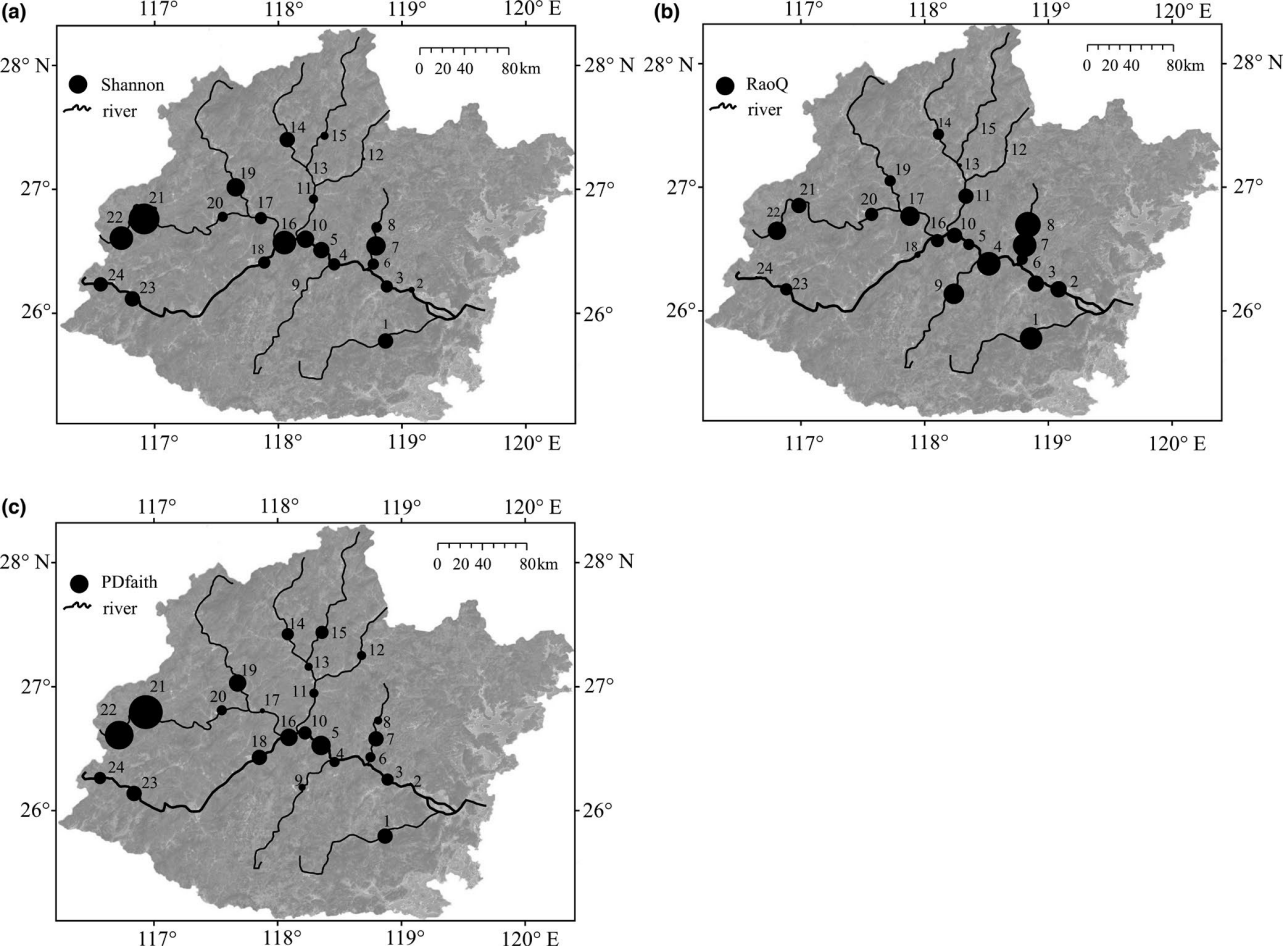
Spatial patterns of alpha diversity in the Min River, including (a) Shannon index (Shannon) as the surrogate for taxonomic diversity, (b) Rao's quadratic entropy (RaoQ) for functional diversity, and (c) Faith's PD index (PDfaith) for phylogenetic diversity

**FIGURE 4:**
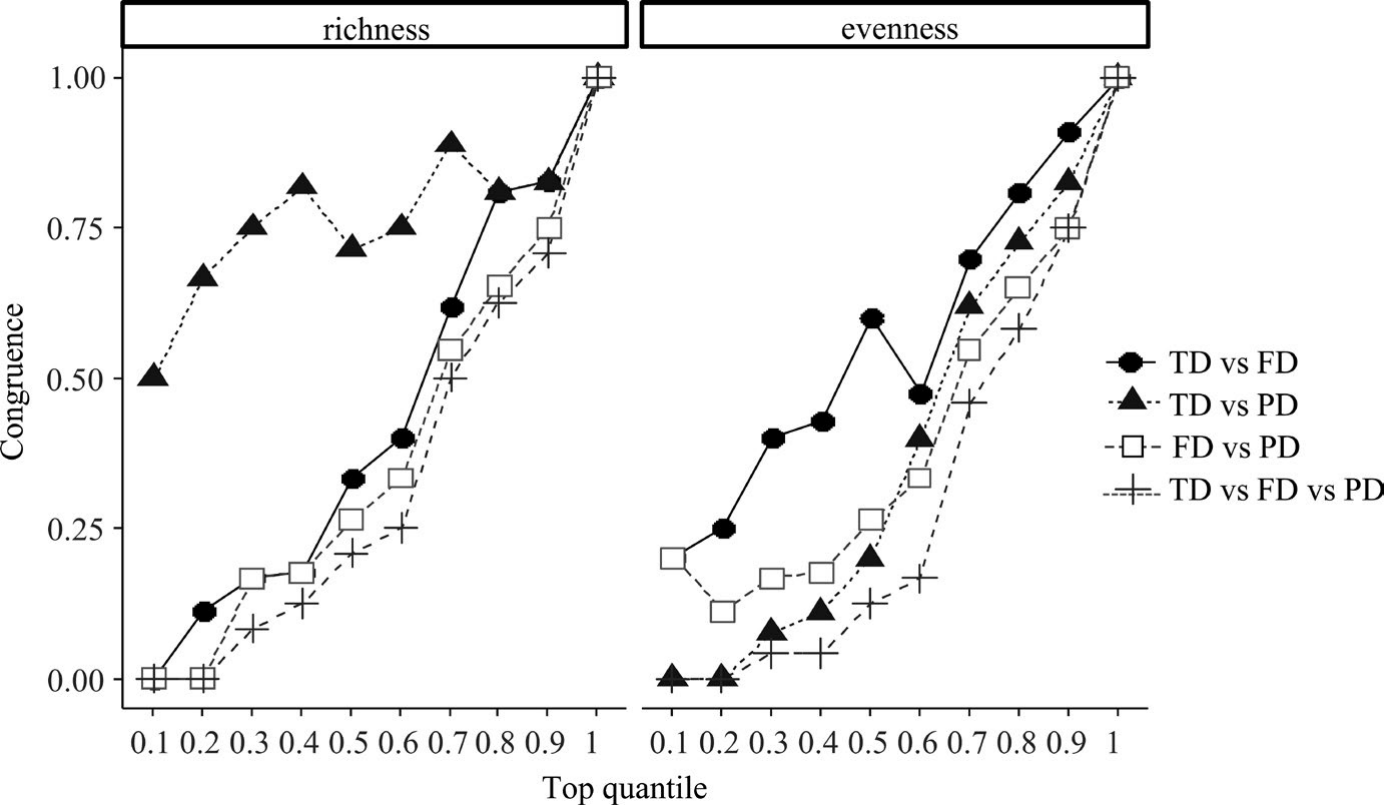
Congruence between alpha richness (left) and evenness (right) of taxonomic diversity (TD), functional diversity (FD), and phylogenetic diversity (PD), where alpha congruence is assessed by comparing the spatial concordance between the top 10% of sampling sites and then successively at 10% intervals. Each line represents the congruence between TD, FD, and PD. For example, the line “TD versus FD” represented the congruence between TD and FD. Surrogates for TD, FD, and PD richness are Shannon index (Shannon), Rao's Quadratic Entropy index (RaoQ), and Faith's PD index (PDfaith), respectively. Surrogates for TD, FD, and PD evenness are Shannon Evenness (SEve), Functional evenness (FEve), and Phylogenetic Species Evenness (PSEve), respectively

Spatial distributions of TD, FD, and PD are shown in Figure 3. TD and PD at Sites 21 and 22 were the highest, whereas FD at Sites 7 and 8 was the highest. Congruence among TD, FD, and PD is shown in Figure 4. Little congruence was found simultaneously among the three facets of biodiversity, neither for richness nor evenness in the first top 10% quantile. Pairwisely, congruence between TD and PD richness in the first top 10% quantile was 50%. Meanwhile, congruence of evenness pairwisely between TD and FD, and FD and PD was around 20%. No other pairwise congruence was observed.

### 3.2 Patterns of beta biodiversity

**FIGURE 5:**
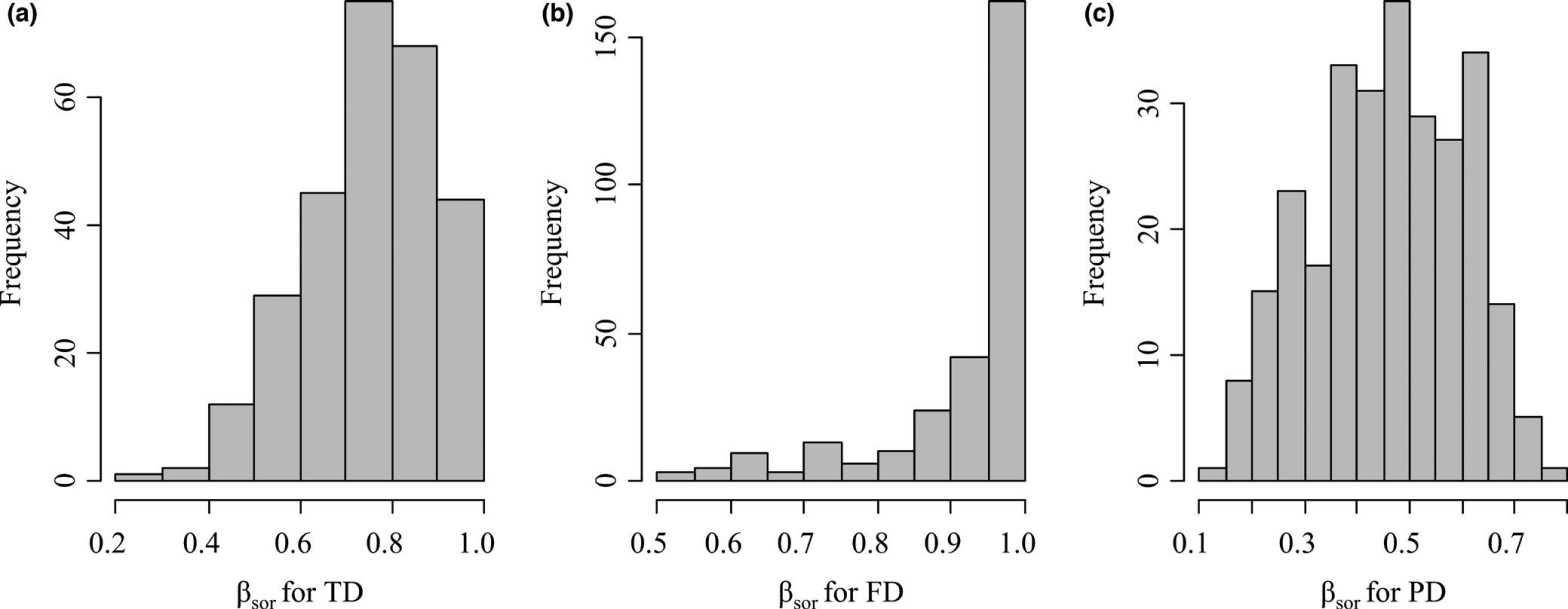
Histograms of beta diversity. (a) Frequency of total taxonomic beta diversity, that is, beta Sorensen index for taxonomic diversity (*β*_sor_ for TD). (b) Frequency of beta Sorensen index for functional diversity (*β*_sor_ for FD). (c) Frequency of beta Sorensen index for phylogenetic diversity (*β*_sor_ for PD)

Values of *β*_sor_ for TD ranged from 0.27 to 1.00 with an average of 0.76 ± 0.15 (Figure 5a), *β*_sor_ for FD ranged from 0.50 to 1.00 with an average of 0.92 ± 0.11 (Figure 5b), and *β*_sor_ for PD ranged from 0.13 to 0.76 with an average of 0.46 ± 0.14 (Figure 5c). The highest *β*_sor_ for TD occurred between Site 2 and Site 24 and the lowest between Site 3 and Site 10, the highest *β*_sor_ for FD occurred between Site 3 and Site 11 and the lowest between Site 21 and Site 22, while the highest *β*_sor_ for PD occurred between Site 8 and Site 10 and the lowest between Site 11 and Site 20.

**FIGURE 6:**
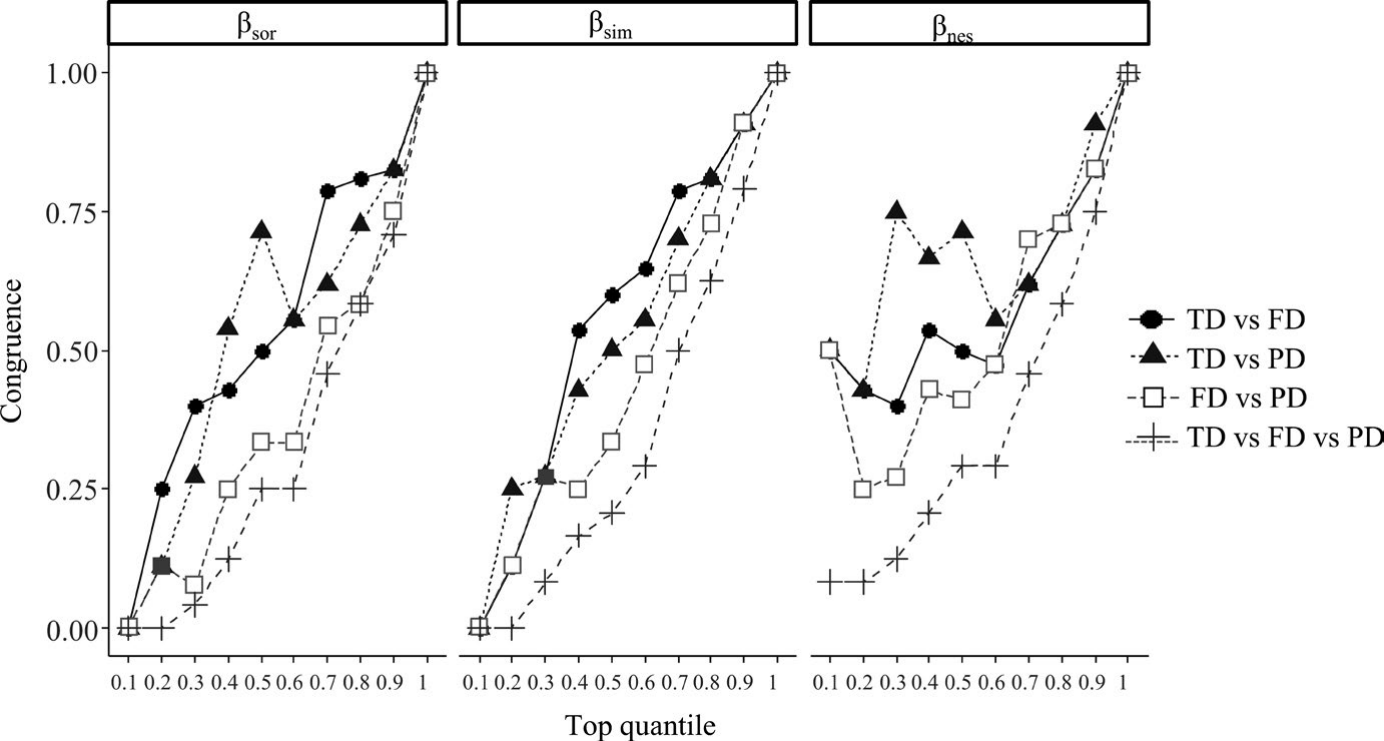
Congruence between Sorensen family beta diversity of taxonomic diversity (TD), functional diversity (FD), and phylogenetic diversity (PD), where the congruence is assessed by comparing the spatial concordance between the top 10% of pairwise beta diversity and then successively at 10% intervals. Each line represents the congruence among beta TD, FD, and PD. For example, the line “TD versus FD” represents the congruence between beta TD and FD

Congruence among beta TD, FD, and PD is shown in Figure 6, including *β*_sor_, *β*_sim_, and *β*_nes_. No *β*_sor_ nor *β*_sim_ congruence was observed in the first top 10% quantile pairwisely among beta TD, FD, and PD. On the other hand, there was about 50% of *β*_nes_ congruence pairwisely and around 10% congruence when simultaneously considering all these three facets of biodiversity.

**FIGURE 7:**
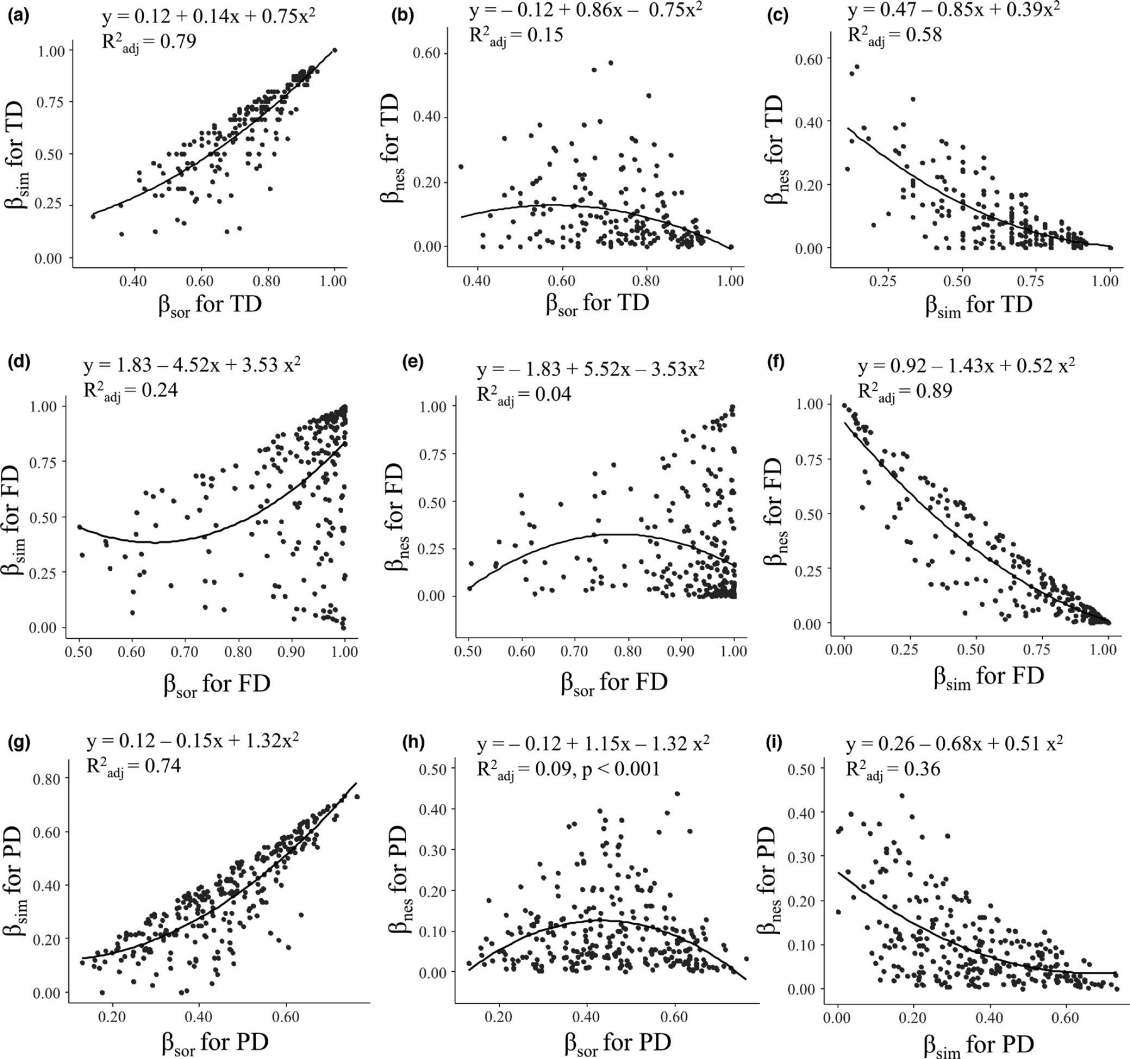
Quadratic regression analysis between beta Sorensen, turnover, and nestedness for different facets of biodiversity, that is, taxonomic diversity (TD) (a, b and c), functional diversity (FD) (d, e and f), and phylogenetic diversity (PD) (g, h and i). The lines represent Ordinary Least Square (OLS) regression lines. In the quadratic least‐squares prediction equations, $R_{\text{adj}}^{2}$ represents the adjusted R square coefficient. “*β*_sor_” represents total beta diversity, that is, beta Sorensen index. “*β*_sim_” and “*β*_nes_” represent two components of *β*_sor_, that is, turnover and nestedness, respectively

Paired regression analysis between *β*_sor_, *β*_sim_, and *β*_nes_ for different biodiversity facets is shown in Figure 7. The relationships between *β*_sor_ and *β*_sim_ were significantly positive for all facets of biodiversity (*p* < .001, Figure 7a,d,g). Meanwhile, the relationships between *β*_sim_ and *β*_nes_ were significantly negative for all facets of biodiversity (*p* < .001, Figure 7c,f,i). No obvious pattern was observed between *β*_sor_ and *β*_nes_.

### 3.3 Effects of spatial pattern of river system

MANOVA testing results of the influence of river Order, log_2_Dlink, and Dam on TD, FD, and PD are shown in Table 4. Dam had a significant influence on TD (*p* < .01), PD (*p* < .001), and FD (*p* < .01). The impacts of Order on TD, FD, and PD were not significant (*p* > .01). Log_2_Dlink had a significant influence on FD (*p* < .01), but not on TD and PD (*p* > .01).

**TABLE 4:** Multivariate analysis of variance (MANOVA) testing results of the influence of stream segment (Order), 2‐based logarithm of downstream link (log_2_Dlink), and existence of hydropower or water reservoir dams (Dam) on Shannon index (Shannon), Rao's Quadratic Entropy index (RaoQ) and Faith's PD index (PDfaith)

Biodiversity	Order		log_2_Dlink		Dam	
	*F* statistics	*p* value	*F* statistics	*p* value	*F* statistics	*p* value
Shannon	0.147	.864	0.993	.449	8.914	.002**
RaoQ	0.414	.666	7.327	.004**	4.256	.098*
PDfaith	1.115	.347	1.371	.281	21.52	8.230e−06***

Note

Significance codes: “***”*p* < .001, “**” indicates *p* < .01, “*” indicates *p* < .01.

**FIGURE 8:**
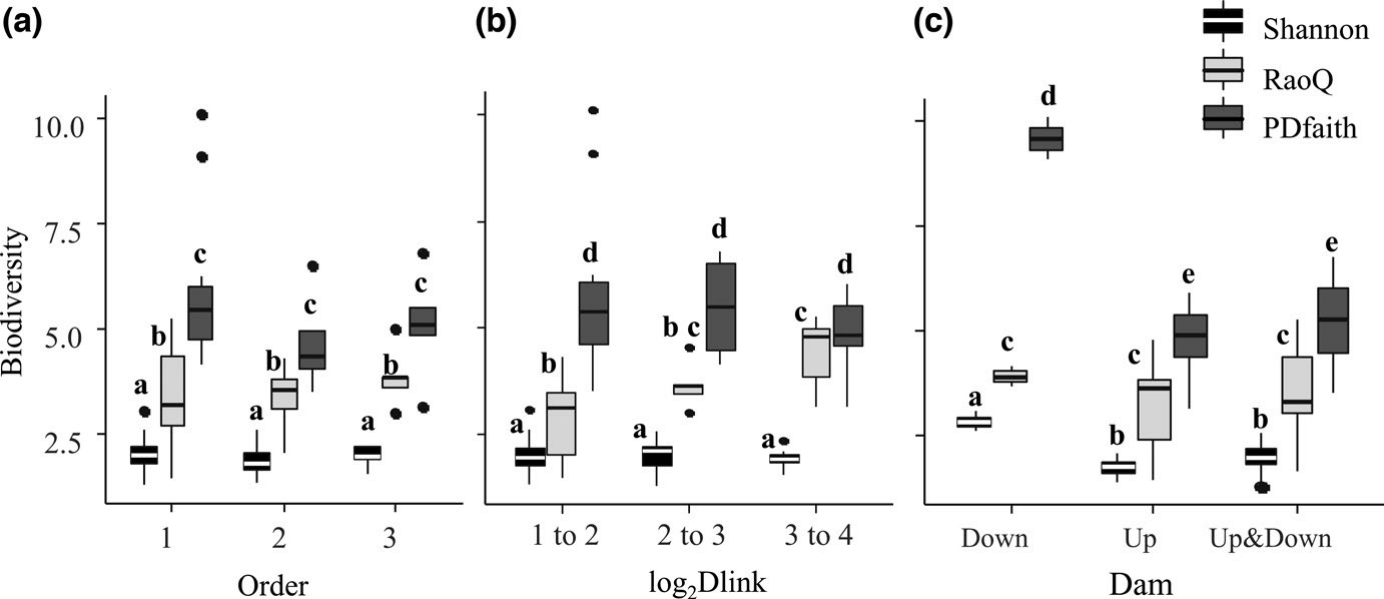
Multivariate analysis of variance analysis (MANOVA) to test the influence of different variables, including Strahler order of stream segment (Order), 2‐based logarithm of downstream link (log_2_Dlink), and existence of dams (Dam), on different biodiversity facets. (a) Test of the influence of Order on different biodiversity facets. 3 stream orders are identified in the Min River. (b) Test of the influence of log_2_Dlink. Categories of the log_2_Dlink are: values from 1 to 2, 2 to 3, and 3 to 4. (c) Test of the influence of Dam. Categories of the Dam are existence of a downstream but no upstream dam (Down), an upstream but no downstream dam (Up), and both upstream and downstream dams (Up&Down). Different lower cases above the box indicate significant differences at the confidence level of 0.99 (*p* < .01). Surrogates for taxonomic diversity, functional diversity, and phylogenetic diversity are Shannon index (Shannon), Rao's Quadratic Entropy index (RaoQ), and Faith's PD index (PDfaith), respectively

Comparisons of different biodiversity facets under different variables are shown in Figure 8. FD at those sites with a categorical log_2_Dlink value of 3–4 was significantly higher than those sites with a categorical log_2_Dlink value of 1–2 (*p* < .01); TD and PD at those sites with the existence of downstream but no upstream dams were significantly higher than those sites with upstream dams (*p* < .01).

### 3.4 Species contributions to diversity vulnerability

Sixteen species that contributed the most (highest 10% quantile on average) to TD, FD, and PD vulnerability were identified (Table 5). All these 16 species possess distinctiveness. Fifteen of them were endemic species. Only species Mozambique tilapia *Oreochromis mossambicus* was exotic species. Ten species among them possessed one or more distinctive traits with the highest or lowest value (highest or lowest 10% quantile). The remaining 6 were all benthopelagic species with the highest occurrence (highest 10% quantile).

**TABLE 5:** Species which contribute the most (highest 10% quantile on average) to biodiversity vulnerability

Species	Family	Top TD	Top FD	Top PD	Endemic (Y/N)	Rheophily	Position of the water column	Highest distinctiveness	Lowest distinctiveness
*Acrossocheilus hemispinus*	Cyprinidae		√		Y	rheophilic	benthopelagic	Highest occurrence	
*Chanodichthys dabryi*	Cyprinidae	√	√		Y	limnophilic	pelagic	Highest occurrence	
*Hemiculter leucisculus*	Cyprinidae	√	√		Y	limnophilic	pelagic	Highest occurrence	Life span
*Microphysogobio fukiensis*	Cyprinidae	√	√		Y	rheophilic	benthic	Life span	
*Opsariichthys bidens*	Cyprinidae	√		√	Y	rheophilic	benthopelagic	Highest occurrence	
*Oreochromis mossambicus*	Cichlidae		√		N	eurytopic	benthopelagic	Hl/Sl; Bd/Sl	Hd/Bd
*Pseudobagrus vachellii*	Bagridae	√			Y	limnophilic	benthic	Hd/Bd	Hl/Sl
*Rhinogobio typus*	Cyprinidae			√	Y	limnophilic	benthic	Snl/Hl	CPd/CFd
*Rhinogobius giurinus*	Gobiidae	√		√	Y	limnophilic	benthopelagic	CPd/CFd	
*Squalidus argentatus*	Cyprinidae	√			Y	rheophilic	benthopelagic	Highest occurrence	
*Squaliobarbus curriculus*	Cyprinidae	√		√	Y	limnophilic	benthopelagic	Highest occurrence	
*Tachysurus fulvidraco*	Bagridae			√	Y	limnophilic	benthic	Highest occurrence	Snl/Hl
*Vanmanenia caldwelli*	Cichlidae		√		Y	rheophilic	benthic	Snl/Hl; Hd/Bd; PecFl/Sl	
*Vanmanenia gymnetrus*	Balitoridae		√		Y	rheophilic	benthic	Snl/Hl; Hd/Bd	Ed/Hd; Bd/Sl
*Xenocypris macrolepis*	Cyprinidae			√	Y	limnophilic	benthopelagic	Hl/Sl	Snl/Hl
*Zacco platypus*	Cyprinidae	√		√	Y	rheophilic	benthopelagic	Highest occurrence	

Note

Symbol “√” indicates that the corresponding species contribute the most on average to specific biodiversity vulnerability. Top TD, FD, and PD indicate top vulnerability of taxonomic, functional, and phylogenetic diversity. Highest or lowest distinctive trait means this species possesses one or more functional traits with the highest or lowest value (highest or lowest 10% quantile), or they are with the highest occurrence (highest 10% quantile). Related traits include Maximum life span in years (Life span), Relative head length (Hl/Sl), Relative body depth (Bd/Sl), Relative head depth (Hd/Bd), relative snout length (Snl/Hl), Swimming factor (CPd/CFd), Relative pectoral fin length, and Relative eye size. Please refer to Table 1 for the biological interpretation of these traits.

### 3.5 Biodiversity vulnerability to species loss

**FIGURE 9:**
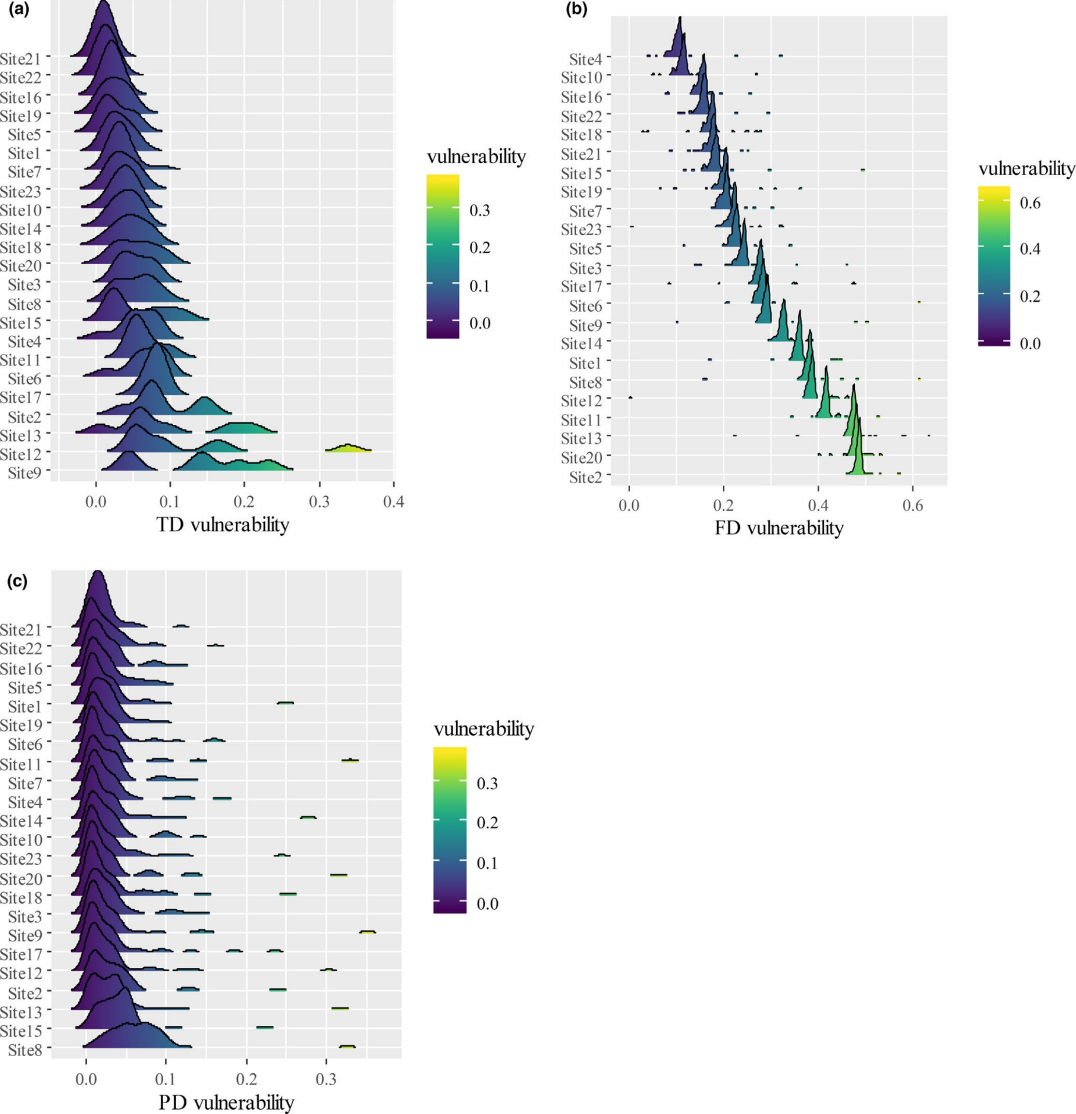
Density plots of diversity vulnerability at different sites, with mean value increasing from top to bottom. (a) Taxonomic diversity (TD) vulnerability in terms of Shannon index. (b) Functional diversity (FD) vulnerability in terms of Rao's Quadratic Entropy (RaoQ). (c) Phylogenetic diversity (PD) vulnerability in terms of Faith's Phylogenetic Diversity (PDfaith)

Density plots of biodiversity vulnerabilities are shown in Figure 9. Variance of FD vulnerability at different sites was significantly higher than those of TD and FD (*p* < .01), indicating that FD was more vulnerable to species loss than TD and FD. Sites 9 and 12 were the most vulnerable in terms of TD, Sites 2 and 20 in terms of FD, and Sites 8 and 15 in terms of PD. Characteristics of these sites are listed in Table 6. At least 3 distinctive species, which were listed in Table 5, were found at these sites. Occupancy of these species (number of distinctive species divided by species richness at this site) was at least 42%. All sites had an upstream dam.

**TABLE 6:** Sites with the highest biodiversity vulnerability (highest 10% quantile)

Site	Vulnerable facet	Species richness	Distinctive species	Occupancy (%)	Dam
Site 9	TD	6	5	83.33	Up & Down
Site 12	TD	9	4	44.44	Up & Down
Site 2	FD	7	3	42.86	Up
Site 20	FD	10	5	50.00	Up & Down
Site 8	PD	10	5	50.00	Up
Site 15	PD	11	6	54.55	Up & Down

Note

Column “Vulnerable facets” indicates that this site is the most vulnerable to species loss in terms of taxonomic diversity (TD), functional diversity (FD), or Phylogenetic diversity (PD). Colum “Distinctive species” refers to the number of distinctive species found at this site. Column “Occupancy” refers to number of distinctive species divided by species richness at this site. Column “Dam” refers to the existence of an upstream but no downstream dam (Up), or both upstream and downstream dams (Up & Down).

## 4 DISCUSSION

This research revealed the spatial patterns of different biodiversity facets in the Min River, explored the impact of spatial pattern of river system, and evaluated the biodiversity vulnerability to species loss. These results are meaningful during the conservation and protection strategy‐making processes in a practical perspective.

### 4.1 Effects of dams on patterns of biodiversity

In this work, existence of dams was significantly correlated with the spatial patterns of different biodiversity facets. Hotspots for TD and PD were Sites 21 and 22, while for FD were Sites 7 and 8. It is noticed that near downstream of these two sites is located a big hydroelectricity station, and impoundment here forms the Chitan Reservoir, with a vast area of 37 km^2^. Impoundment caused by dam construction imposed varieties of ecological effects on fishes (Franssen & Tobler, 2013; Mbaka & Mwaniki, 2015). New lentic habitat was formed, water depth rises, and previous terrestrial areas were inundated. The initial decomposition of terrestrial vegetation intrigued an abundant flux of nutrients. Aquatic primary production is then boosted, and some trophic groups prosper both in terms of species abundance and richness (Arantes et al., 2019). These trophic groups often included some detritivores, herbivores, omnivores, and invertivores, which supported higher TD and PD in the reservoir at the early stage (Miranda et al., 2019; Turgeon et al., 2016). For example, 23 out of 31 fish species collected at Site 21 belong to these groups, and the most abundant fishes are detritivorous species Freshwater minnow *Zacco platypus* and omnivorous species *Acrossocheilus paradoxus*.

However, high TD and PD in an impounded reservoir do not necessarily support high FD in the long run. For example, an increase in species richness and abundance but decrease in functional diversity was observed in the Segura River in southern Spain (Sanchez‐Perez et al., 2020). In the upstream of the dam after the formation of a reservoir, the rise of water depth stratifies dissolved oxygen because of a vertical water pressure gradient. Thus, benthic fishes lose their habitat suitability as dissolved oxygen is depleted in the bottom water column (Yang et al., 2020). Also, lentic reservoir environment is not suitable for rheophilic fishes. Functional trait groups, such as dorso‐ventrally compressed bodies, inferior mouths, and reduced swim bladders, are diminished (Arantes et al., 2019). In this work, similar effects of functional trait filtering had been observed. For instance, species lesser spiny eel *Macrognathus aculeatus* possesses the smallest value of swimming factor CPd/CFd (caudal peduncle depth divided by caudal fin depth), which indicates that it is adaptive to the lotic water environment in the Min River. It was found at Site 20, downstream of the Chitan Reservoir, but absent from Site 21, upstream of the dam. On the other hand, the highest FD was observed at Sites 7 and 8, where no big dams were constructed along the main course of this tributary. This probably explained that even though Sites 21 and 22 were hotspots for TD and PD, they did not support equivalent levels of FD.

Connectivity of the river is critical for fishes to prosper because they need to move along watercourses to reach suitable habitats (Griffiths, 2017). The connectivity is always fragmented by the construction of dams, which is one of the most hazardous factors (Wang et al., 2011). For example, dams in the Danube River in Europe drive the loss of habitat suitability for some endangered species (Brinker et al., 2018). The same effects might have also occurred in the Min River. There were over 150 fish species in this basin in the 1970s (Lian, 1988; Zhu, 1984), but only 82 were collected in this work. According to local official annals, there had been over 3,800 hydropower stations and water reservoirs of different sizes in Fujian Province by the year 2010, many of which were located in the Min River (Annals, 1999). Thus, the protection of freshwater biodiversity and economic development should be balanced so that fishes will survive under pressures imposed by anthropogenic activities in the Min River.

Of course, many factors contribute to shaping the patterns of biodiversity (Graham et al., 2005; López‐Delgado et al., 2020; Olah et al., 2016). In future studies, incorporation of abiotic factors (such as temperature, physical water source, water pollution, and water velocity) and biotic factors (such as anthropogenic activities, interspecific interactions, species invasion, and species dispersibility) to discuss the effects of these factors on biodiversity will further clarify the spatial pattern of biodiversity.

### 4.2 Incongruence of different biodiversity facets

During the past decades, there have been multiple works trying to seek congruence of different biodiversity facets (Gonzalez‐Maya et al., 2016; Roa‐Fuentes et al., 2019; Strecker et al., 2011). The best scenario is that all facets of biodiversity are highly congruent over space (Strecker et al., 2011). For example, hotspots of TD, FD, and PD were highly congruent in native freshwater fish communities in France (Pool et al., 2014). Indeed, TD, FD, and PD are somewhat correlated because they are all derived from the distribution information of the species which assemble the community. However, many abiotic or biotic factors as well as random processes, as mentioned above, holistically contribute to the community assembly process. Correlations between different facets of biodiversity are weakened by these factors. Thus, incongruences occurred in many cases on regional scales, and it raised the challenge that trade‐offs were inevitable when protecting multiple biodiversity facets (Doxa et al., 2020; Kuczynski et al., 2018). This is the case in the Min River, whose mismatches of different facets of biodiversity were correlated with segmented river networks.

There are alternative methods which do not need all different biodiversity facets to agree (Cadotte & Tucker, 2018). Some well‐developed algorithms including *Zonation*, *Marxan*, and commercial computation program *Gurobi* are conservation decision supporting tools for prioritization (Helm & Justkowiak, 2018; Moilanen, 2005; Watts et al., 2009). Although they differ in underlying algorithms, they have some attributes in common (Delavenne et al., 2012): (a) They attempt to maximize conservation targets through setting a priori features, (b) they seek a solution to minimize conservation cost while ensuring those conservation targets are met, (c) and they need real‐world species distribution data. These algorithms have a plethora of applications nowadays. But they also have some limitations for end‐users: (a) Although their algorithms are clear in the handbook, calculation of irreplaceability or vulnerability tends to be a black box; (b) some features are choosy, and (c) some data are barely available, such as conservation cost per unit area and species distribution data in a fine grid cell resolution. Conservation cost is mostly substituted by human population data in some cases because the denser the population, the more costly to purchase land for conservation. Meanwhile, species distribution data mostly rely on niche models, which is another hot topic nowadays.

Environmental, social‐economical, and cultural factors should be considered when applying these systematic conservation algorithms (Delavenne et al., 2012; Isotti & Monacelli, 2019; Ma et al., 2020). Functional distinctiveness, phylogenetic endemism, ecological services, vegetation, and cultural preferences could be weighted as conservation features (Ainsworth et al., 2018; Jiang et al., 2020; Kosman et al., 2019; Ma et al., 2021). Given that scarce efforts have been deployed in fish conservation in the Min River, this work will help to develop systematic conservation planning in the following ways: (a) Species distribution data in this work will help with further species distribution modeling, especially for those endemic species whose distribution is hardly found in other literature; (b) the finding that existence of dams is most possibly the driving factor for patterns of biodiversity will help to select important features for prioritization; and (c) the spatial patterns of TD, FD and PD will help with the validation of the effectiveness of systematic conservation planning in practice.

Incongruence of different biodiversity facets does not impair the necessity to protect hotspots of TD, FD, and PD. Because of a limited budget, and that there have been few conservation researches regarding this river so far, available information was not enough to scheme a complete systematic conservation planning for the whole basin. Nevertheless, as the conservation in the Min River is imminent, it should be considered to start fish biodiversity conservation at hotspots of TD, FD, and PD suggested in this work before more feasible systematic conservation planning has been composed, that is, Sites 21 and 22 for TD and PD, and Sites 7 and 8 for FD.

### 4.3 Species prioritization based on species contributions

Species protection is an important task for conservation (Guilhaumon et al., 2015; Loiseau et al., 2017). At least some minimum number of species were essential for ecosystem functioning under constant conditions and that a larger number of species were probably essential for maintaining the stability of ecosystem processes in changing environments (Loreau et al., 2001; Wilson et al., 2005). Some researchers placed more emphasis on distinctive species, as species with more distinctiveness are supposed to be more important in shaping spatial patterns of biodiversity in most cases (Cooke et al., 2020). It was also reported that species with higher prevalence were mainly responsible for spatial patterns of TD in segmented environments (Bregovic et al., 2019). Some conservation prioritized endemic species over exotic species, as exotic species often outcompete local species and cause biodiversity homogenization (Milardi et al., 2019). All those 16 species that contributed the most to biodiversity vulnerability in this work possess distinctiveness. They either carry distinctive functional traits with extreme values or they are prevalent species with the highest occurrence. For example, species *Hemiculter leucisculus* is distinctive with the highest values of relative snout length, relative head depth, and relative pectoral fin length. These results highlight those species in the Min River from the perspective of prevalence, endemism, or functional distinctiveness. Thus, these species are probably worthy of conservation interest in conservation prioritization.

Species Mozambique tilapia *Oreochromis mossambicus* is an invasive species in the Min River (Deng et al., 2020). It is highly commercial and widely introduced for aquaculture (Froese & Pauly, 2019). As a most successful and vagile invader, it often outcompetes local species (Kottelat & Whitten, 1996). Thus, it was reported as an adverse ecological factor in several countries (Russell et al., 2012). Its contribution to FD vulnerability indicates that this species probably have successfully colonized the Min River. It is important to emphasize that further investigations and researches regarding the influence of its invasion on the local biodiversity are still indispensable. However, conservation efforts must take this species into precautious consideration because invasive alien species always trigger biodiversity homogenization (Milardi et al., 2019).

### 4.4 Sites prioritization based on biodiversity vulnerability

Biodiversity vulnerability is another facet that should be taken into consideration during the conservation prioritization process (Bellard et al., 2014). Areas inhabited by species which are responsive to environmental changes always show more sensitivity to biodiversity loss (Heilpern et al., 2018). Sites with distinctive species were most vulnerable to species loss in the Min River. For example, 5 out of 6 species at Site 9 possessed the highest vulnerability for TD.

All sites with the highest vulnerability were located downstream of a dam in the Min River. Impoundment caused by a dam reduces habitat suitability for some upstream fishes, while other processes could occur for downstream fishes. Water discharge and velocity show seasonality in the Min River (Editorial Committee of Fujian Province Annals, 1999). Out of the flood season, downstream water is shallow, water velocity and discharge are both reduced, and sometimes the riverbed is even depleted of visible water. Fishes adaptive to both fast‐flowing water and lentic environment lose their chances of survival. On the other hand, water velocity and discharge exceed the limit that one species could tolerate in the flood season, and most fishes will be flushed away from the dam. Meanwhile, an upstream dam creates a physical barrier for migratory fish species, which leads to a reduction in their population, even extinction (Wang et al., 2011). That is probably one of the reasons why FD was the most vulnerable compared with TD and PD in this work. From this perspective, besides conserving those hotspots for TD, FD, and PD, prioritizing those sites with the highest functional diversity vulnerabilities in the Min River, that is, Sites 2 and 20, should be considered.

## CONFLICT OF INTEREST

None declared.

## AUTHOR CONTRIBUTIONS

**Li Lin:** Conceptualization (equal); data curation (equal); formal analysis (lead); methodology (equal); resources (equal); software (lead); validation (equal); visualization (lead); writing–original draft (lead). **Weide Deng:** Conceptualization (equal); data curation (equal); investigation (lead); project administration (equal); resources (equal); validation (equal). **Xiaoxia Huang:** Conceptualization (equal); methodology (equal); project administration (equal); resources (equal); software (equal). **Bin Kang:** Conceptualization (equal); funding acquisition (lead); methodology (equal); project administration (equal); supervision (equal); validation (equal); writing–review and editing (lead).

## Supporting information

Supplementary Material

## Data Availability

The fish species abundance matrix, fish functional traits, and GenBank accession numbers for CYTB and COI genes are stored in Dryad repository (https://doi.org/10.5061/dryad.g1jwstqrf). Additional information, including R scripts, will also be disclosed via emails to our corresponding authors at the request of readers.
