# Supplementary material for: Fish taxonomic, functional, and phylogenetic diversity and their vulnerabilities in the largest river in southeastern China
Source: Ecol Evol. 2021 Jul 24;11(16):11533–48. doi: 10.1002/ece3.7945 (PMC8366846; doi:10.1002/ece3.7945)
Supplement: Supplementary file 1 — Supplementary Material [file ECE3-11-11533-s001.docx]

**SUPPORTING INFORMATION**

**Table S1** Fishes collected and their occurrences across 24 sample sites in the Min River. Sampling was carried out in May 2015.

| Species | Family | Total abundance | Sites occupied |
| --- | --- | --- | --- |
| *Abbottina rivularis* | Cyprinidae | 33 | 5 |
| *Acheilognathinae sp.* | Cyprinidae | 1 | 1 |
| *Acheilognathus barbatulus* | Cyprinidae | 1 | 1 |
| *Acheilognathus barbatus* | Cyprinidae | 1 | 1 |
| *Acrossocheilus hemispinus* | Cyprinidae | 93 | 7 |
| *Acrossocheilus kreyenbergii* | Cyprinidae | 2 | 2 |
| *Acrossocheilus paradoxus* | Cyprinidae | 35 | 2 |
| *Acrossocheilus parallens* | Cyprinidae | 1 | 1 |
| *Acrossocheilus wenchowensis* | Cyprinidae | 4 | 2 |
| *Anguilla japonica* | Anguillidae | 3 | 2 |
| *Belligobio nummifer* | Cyprinidae | 4 | 1 |
| *Carassius auratus* | Cyprinidae | 31 | 7 |
| *Channa maculata* | Channidae | 7 | 3 |
| *Chanodichthys dabryi* | Cyprinidae | 33 | 7 |
| *Chanodichthys erythropterus* | Cyprinidae | 1 | 1 |
| *Cobitis taenia* | Cobitidae | 36 | 6 |
| *Ctenopharyngodon idella* | Cyprinidae | 4 | 4 |
| *Culter alburnus* | Cyprinidae | 19 | 7 |
| *Culterinae sp.* | Cyprinidae | 5 | 1 |
| *Cyprinus carpio* | Cyprinidae | 4 | 4 |
| *Distoechodon tumirostris* | Cyprinidae | 45 | 5 |
| *Glyptothorax fokiensis* | Sisoridae | 4 | 2 |
| *Gobiidae sp.1* | Gobiidae | 5 | 1 |
| *Gobiidae sp.2* | Gobiidae | 2 | 1 |
| *Gobiidae sp.5* | Gobiidae | 24 | 3 |
| *Gobiidae sp.6* | Gobiidae | 4 | 2 |
| *Hemibagrus macropterus* | Bagridae | 4 | 2 |
| *Hemibarbus maculatus* | Cyprinidae | 7 | 3 |
| *Hemiculter leucisculus* | Cyprinidae | 114 | 13 |
| *Lateolabrax japonicus* | Lateolabracidae | 4 | 1 |
| *Lepomis gulosus* | Centrarchidae | 1 | 1 |
| *Liobagrus anguillicauda* | Amblycipitidae | 3 | 1 |
| *Macrognathus aculeatus* | Mastacembelidae | 3 | 2 |
| *Macropodus opercularis* | Osphronemidae | 17 | 3 |
| *Megalobrama amblycephala* | Cyprinidae | 2 | 1 |
| *Megalobrama terminalis* | Cyprinidae | 5 | 2 |
| *Microphysogobio fukiensis* | Cyprinidae | 97 | 10 |
| *Microphysogobio kiatingensis* | Cyprinidae | 6 | 3 |
| *Misgurnus anguillicaudatus* | Cobitidae | 26 | 4 |
| *Monopterus albus* | Synbranchidae | 2 | 2 |
| *Onychostoma barbatulum* | Cyprinidae | 15 | 2 |
| *Opsariichthys bidens* | Cyprinidae | 101 | 13 |
| *Oreochromis mossambicus* | Cichlidae | 40 | 4 |
| *Pseudobagrus albomarginatus* | Bagridae | 1 | 1 |
| *Pseudobagrus crassilabris* | Bagridae | 1 | 1 |
| *Pseudobagrus tenuis* | Bagridae | 5 | 4 |
| *Pseudobagrus truncatus* | Bagridae | 11 | 3 |
| *Pseudobagrus vachellii* | Bagridae | 26 | 8 |
| *Pseudogastromyzon fasciatus* | Balitoridae | 8 | 3 |
| *Pseudogobio vaillanti* | Cyprinidae | 4 | 2 |
| *Pseudohemiculter dispar* | Cyprinidae | 30 | 5 |
| *Pseudolaubuca sinensis* | Cyprinidae | 15 | 9 |
| *pseudorasbora parva* | Cyprinidae | 4 | 3 |
| *Pterocryptis cochinchinensis* | Siluridae | 1 | 1 |
| *Puntius semifasciolatus* | Cyprinidae | 10 | 2 |
| *Rhinogobio typus* | Cyprinidae | 26 | 7 |
| *Rhinogobius giurinus* | Gobiidae | 118 | 15 |
| *Rhodeus ocellatus* | Cyprinidae | 5 | 1 |
| *Rhodeus sinensis* | Cyprinidae | 17 | 3 |
| *Sarcocheilichthys kiangsiensis* | Cyprinidae | 1 | 1 |
| *Sarcocheilichthys parvus* | Cyprinidae | 2 | 2 |
| *Sarcocheilichthys sinensis* | Cyprinidae | 9 | 2 |
| *Saurogobio dabryi* | Cyprinidae | 12 | 3 |
| *Silurus asotus* | Siluridae | 3 | 3 |
| *Sinibotia superciliaris* | Cobitidae | 3 | 1 |
| *Sinibrama macrops* | Cyprinidae | 38 | 6 |
| *Sinibrama wui* | Cyprinidae | 3 | 1 |
| *Siniperca chuatsi* | Percichthyidae | 1 | 1 |
| *Siniperca knerii* | Percichthyidae | 4 | 2 |
| *Siniperca scherzeri* | Percichthyidae | 1 | 1 |
| *Siniperca undulata* | Percichthyidae | 2 | 2 |
| *Spinibarbus caldwelli* | Cyprinidae | 16 | 4 |
| *Squalidus argentatus* | Cyprinidae | 59 | 8 |
| *Squaliobarbus curriculus* | Cyprinidae | 37 | 10 |
| *Tachysurus adiposalis* | Bagridae | 1 | 1 |
| *Tachysurus fulvidraco* | Bagridae | 17 | 7 |
| *Tanakia himantegus* | Cyprinidae | 1 | 1 |
| *Vanmanenia caldwelli* | Balitoridae | 56 | 5 |
| *Vanmanenia gymnetrus* | Balitoridae | 30 | 7 |
| *Xenocypris davidi* | Cyprinidae | 41 | 6 |
| *Xenocypris macrolepis* | Cyprinidae | 65 | 7 |
| *Zacco platypus* | Cyprinidae | 270 | 14 |

**Table S2.** Main R packages and corresponding functions applied in this work.

| **R package** | **Function** | **Specific action** |
| --- | --- | --- |
| BiodiversityR | diversityresult | Alfa TD calculation. |
| FD | dbFD | FD indices calculation. |
| ape | clustal | Sequence alignment with Clustal W 2.0. |
| ape | Muscle | Sequence alignment with MUSCLE 3.8. |
| ape | phymltest | Optimal parameters for the Maximum Likelihood phylogenesis. |
| phangorn | pml | Maximum Likelihood tree construction. |
| picante | psd | Alfa PD calculation. |
| vegan | pd | Faith's PD calculation. |
| betapart | beta.pair | Sorensen family beta diversity calculation. |
| ade4 | cmdscale | Principal coordinates analysis. |
| ggplot2 | ggplot | Data visualization. |
| base | scale | Data standardization. |

**Figure S1.** Basic morphological traits measurement for the fish. Sl: Standard length; Hl: Head length; PecFl: Pectoral fin length; Snl: Snout length; Bd: Body depth; Hd: Head depth; Ed: Eye diameter; CPd, Caudal peduncle depth; CFd: Caudal fin depth.

**Figure S2.** Akaike Information Criterion (AIC) for Maximum Likelihood (ML) phylogenetic analysis. The smaller the AIC is, the better that the substitution model explains the empirical data (Posada & Crandall, 2001). The optimum substitution model “GTR + I + Γ” is the one that best explains the empirical data in terms of AIC, where “GTR” denotes the general time-reversible model, meanwhile "+I" and "+G" indicates that invariant sites and a gamma distribution of substitution rates have been specified.
